# *In vivo* and *in vitro* protective effects of shengmai injection against doxorubicin-induced cardiotoxicity

**DOI:** 10.1080/13880209.2022.2046801

**Published:** 2022-03-17

**Authors:** Peng Zhou, Ge Gao, Chun-chun Zhao, Jing-ya Li, Jian-fei Peng, Shu-shu Wang, Rui Song, Hui Shi, Liang Wang

**Affiliations:** a School of Integrated Chinese and Western Medicine, Anhui University of Chinese Medicine, Hefei, China; b Institute of Integrated Chinese and Western Medicine, Anhui Academy of Chinese Medicine, Hefei, China; c Anhui Province Key Laboratory of Chinese Medicinal Formula, Hefei, China; d Nursing School, Anhui University of Chinese Medicine, Hefei, China

**Keywords:** Nrf2/Keap1 signal pathway, apoptosis, oxidative, DOX

## Abstract

**Context:**

Shengmai injection (SMI) has been used to treat heart failure.

**Objective:**

This study determines the molecular mechanisms of SMI against cardiotoxicity caused by doxorubicin (DOX).

**Materials and methods:**

*In vivo*, DOX (15 mg/kg) was intraperitoneally injected in model, Dex (dexrazoxane), SMI-L (2.7 mL/kg), SMI-M (5.4 mL/kg), and SMI-H (10.8 mL/kg) for 7 consecutive days. Hematoxylin-eosin (HE) and Masson staining were used to evaluate histological changes, and cardiomyocyte apoptosis was identified using TdT-mediated dUTP nick-end labelling (TUNEL). Enzymatic indexes were determined. mRNA and protein expressions were analysed through RT-qPCR and Western blotting. *In vitro*, H9c2 cells were divided into control group, model group (2 mL 1 μM DOX), SMI group, ML385 group, and SMI + ML385 group, the intervention lasted for 24 h. mRNA and protein expressions were analysed.

**Results:**

SMI markedly improved cardiac pathology, decreased cardiomyocyte apoptosis, increased creatine kinase (CK), lactate dehydrogenase (LDH), malondialdehyde (MDA), decreased superoxide dismutase (SOD). Compared with the model group, the protein expression of nuclear factor erythroid2-related factor 2 (Nrf2) (SMI-L: 2.42-fold, SMI-M: 2.67-fold, SMI-H: 3.07-fold) and haem oxygenase-1(HO-1) (SMI-L: 1.64-fold, SMI-M: 2.01-fold, SMI-H: 2.19-fold) was increased and the protein expression of kelch-like ECH-associated protein 1 (Keap1) (SMI-L: 0.90-fold, SMI-M: 0.77-fold, SMI-H: 0.66-fold) was decreased in SMI groups and Dex group *in vivo*. Additionally, SMI dramatically inhibited apoptosis, decreased CK, LDH and MDA levels, and enhanced SOD activity. Our results demonstrated that SMI reduced DOX-induced cardiotoxicity via activation of the Nrf2/Keap1 signalling pathway.

**Conclusions:**

This study revealed a new mechanism by which SMI alleviates DOX-induced 45 cardiomyopathy by modulating the Nrf2/Keap1 signal pathway.

## Introduction

Doxorubicin (DOX) is an effective antitumor drug commonly used to treat a variety of cancers, including leukaemia, neuroblastoma, and breast cancer (Zhu et al. 2020). However, its dose-dependent cardiotoxicity often leads to myocardial systolic dysfunction, dilated cardiomyopathy and even heart failure, thus hindering its clinical application (Sawicki et al. 2021). Due to the important role of DOX in tumour chemotherapy, many studies have focussed on exploring the molecular mechanisms by which DOX reduces cardiotoxicity (Gu et al. 2021). At present, dexrazoxane is the only protective agent approved for the prevention and treatment of DOX-induced cardiotoxicity, but it has a serious risk of myelosuppression (Dallons et al. 2020; Zhang et al. 2021). Chinese medicine has the advantages of wide application, few side effects, accurate efficacy and high-cost performance, as well as features of multi-component, multi-pathway and multi-target comprehensive intervention, which has great potential in the prevention and treatment of cardiotoxicity caused by DOX (Huang et al. 2019; Li et al. 2020).

Shengmai injection (SMI), a traditional Chinese medicine extracted from Shengmai san, includes *Panax ginseng* C.A. Mey., *Ophiopogon japonicus* (Thunb.) Ker Gawl., and *Schisandra chinensis* (Turcz.) Baill. (Wang et al. 2020). SMI is widely applied to the treatment of myocardial infarction, myocardial ischaemia-reperfusion, heart failure, diabetic cardiomyopathy and myocarditis, which is related to its anti-lipid peroxidation properties, scavenging oxygen free radicals, anti-ischaemia and hypoxia and protecting cardiomyocytes (Zhu et al. 2019; Zhang et al. 2021). SMI has been shown in a previous study to greatly reduce endothelial cell glycoprotein envelope loss, improve heart function after reperfusion, and reduce oxidative stress (Chen et al. 2021). SMI can effectively promote angiogenesis and reduce ventricular remodelling after ischaemia reperfusion, thus achieving cardiac protection (Liu et al. 2018). SMI could also alleviate myocardial endoplasmic reticulum stress (ERS) and caspase-12 dependent apoptosis, which subsequently helped to improve the heart function of rats with doxorubicin-induced cardiomyopathy (Chen et al. 2015). SMI suppressed Ang II-induced cardiomyocyte hypertrophy and apoptosis via activation of the AMP-activated protein kinase (AMPK) signalling pathway through energy-dependent mechanisms (Li et al. 2019). However, the precise cellular mechanism responsible for the protective effect of the DOX-induced cardiotoxicity remains poorly known.

Thus, the present study explores the role of SMI *in vivo* and *in vitro*, identifies the potential targets in clinical treatment, and elucidates the underlying molecular mechanisms with a focus on the nuclear factor erythroid2-related factor 2 (Nrf2)/kelch-like ECH-associated protein 1 (Keap1) signalling pathway in DOX-induced rat model.

## Materials and methods

### Molecular docking

Previous studies found that there were 11 chemical components of SMI in rat serum, ginsenoside Rg1, Re, Rf, Rg2, Rb1, Rd, Rc, ophiopogonin D, schisandrin, schisandrol B, and schizandrin B (Zhan et al. 2015). CB-DOCK was used to predict protein cavities, calculate the centre and size of the cavities, and finally conduct molecular docking between the main components of SMI and targets. PDB formats of Keap-1 (PDB Code: 4L7B) (Jnoff et al. 2014), and the main components of SMI in SDF format were input to CB-Dock (http://clab.labshare.cn/cb-dock/php/blinddock.php) for molecular docking (Liu et al. 2020). The style of ligand was set as “spacefill”, and the target was set as “cartoon”. The lower the VINA score means the stronger the binding ability between ligand and target.

### Animals

Sprague-Dawley (SD) rats (body weight = 200 ± 20 g) were purchased from the Experimental Animal Centre of Anhui Medical University [Certificate No. SCXK 2017-001]. All experiments met the requirements pertaining to the handling of animals and thus were approved by the Experimental Animal Ethics Committee of Anhui University of Chinese Medicine (Protocol number: AHUCM-rats-2021077).

### Chemicals and regents

SMI (Batch number: 20180620) was purchased from Yisheng Pharmaceutical Co., Ltd. (Jilin Province, China), dexrazoxane (National code: H20061157, Batch number: ABH5564) was purchased from Jiangsu Aosaikang Pharmaceutical Co., Ltd. (Jiangsu Province, China), and doxorubicin hydrochloride (Batch number: RH208406) was purchased from Shanghai Yien Chemical Technology Co., Ltd. (Shanghai, China). ML385 (Batch number: RH203510) was purchased from AbMole company. Nrf2, Keap-1, HO-1 and β-actin were purchased from Abcam (USA) and those against caspase-3, Bcl-2 and Bax were from Abbkine (China). The H9c2 cell line was purchased from the cell resource centre of Shanghai Institute of Life Sciences, Chinese Academy of Sciences.

### *In vivo* experiment

#### Groups of rats and drug treatment

After 1 week of adaptive feeding at a constant temperature, 60 rats were randomly divided into normal group (normal saline), model group (doxorubicin, 15 mg/kg), Dex group (dexrazoxane, 150 mg/kg), SMI-L group (2.7 mL/kg), SMI-M group (5.4 mL/kg), and SMI-H group (10.8 mL/kg), with 10 rats in each group. Normal and model groups were intraperitoneally injected with normal saline every day for 7 consecutive days. The Dex group was intraperitoneally injected with dexrazoxane (150 mg/kg, 10:1 dosage ratio to DOX according to the instructions) on day 7 (Chen et al. 2017). SMI-L, SMI-M, and SMI-H groups were intraperitoneally injected for 7 consecutive days. On day 7, after intraperitoneal injection of drugs, DOX (15 mg/kg) was intraperitoneally injected 30 min later, and the survival status of rats in each group was observed (Prathumsap et al. 2020). Fourty-eight hours after DOX injection, the rats were anaesthetised with pentobarbital sodium, blood was taken under sterile conditions, then the heart was dissected and stored in −80 °C refrigerator and 4% paraformaldehyde, respectively.

### Myocardial histopathology

Paraffin was used to embedded 2 mm thick slices of the ventricle and then cut into 5 μm thick sections. Hematoxylin-eosin (HE) staining and Masson staining were used to observe the pathological changes of myocardial structure to determine the therapeutic effect of drugs.

### TUNEL staining

As with the embedding and sectioning procedures of HE staining, sections (thickness = 5 μm) were obtained. The free 3′-OH chain breaks induced by DNA degradation were detected by the TdT-mediated dUTP nick-end labelling (TUNEL) technique. Under the microscope, five visual fields were randomly selected from each slice; we counted the numbers of all cardiomyocytes and of those apoptotic. The apoptosis rate = number of apoptotic cardiomyocytes/number of all cardiomyocytes × 100%.

### Determination of serum enzymology parameters in rat serum

All procedures for creatine kinase (CK), lactate dehydrogenase (LDH), malondialdehyde (MDA) content, and superoxide dismutase (SOD) activity detection were performed in strict accordance with the kit instructions. In this experiment, the activity of SOD in myocardial tissue was determined using the xanthine oxidase method. The content of CK and LDH in myocardial tissue was determined using the micro-enzyme method. The content of MDA in myocardial tissue was determined using the thiobarbital method.

### RT-PCR for mRNA expression

Total RNA was extracted from myocardial tissue at 50 mg using the Trizol reagent and then reverse-transcribed to cDNA. The cDNA was then analysed using ABI 7500 real-time fluorescent quantitative PCR instrument (ABI, USA). Reaction conditions were set as follows: (1) 95 °C for 5.5 min, (2) 95 °C for 9 s, (3) 60 °C for 29 s. After 40 cycles of amplification, mRNA expression levels of Nrf2, Keap1 and HO-1 were analysed using the comparative 2^-ΔΔCt^ method. The primer sequences used are shown in Table 1.

**Table 1.** Primers used in RT-qPCR.

Primers	Sequence (5′→3′)	
Nrf2	Forward	TTTGTAGATGACCATGAGTCGC
	Reverse	TGTCCTGCTGTATGCTGCTT
Keap1	Forward	CTTCGGGGAGGAGGAGTTCT
	Reverse	GGGCAGTCGTATTTGACCCA
HO-1	Forward	TCTGCAGGGGAGAATCTTGC
	Reverse	TTGGTGAGGGAAATGTGCCA
β-Actin	Reverse	AAGAGGGATGCTGCCCTTAC
	Reverse	ATCCGTTCACACCGACCTTC

### Western blot for protein expression

The concentration of total protein isolated from the myocardium was measured by the bicinchoninic acid method. The proteins were separated using SDS-PAGE and transferred to a nitrocellulose membrane (PVDF, Merck Millipore Ltd., USA). The proteins were blocked in 5% non-fat dry milk for 2 h and incubated overnight at 4 °C. The concentrations of antibodies were as follows: Nrf2 (1:1,000), Keap1 (1:1,000), HO-1 (1:1,000), and β-actin (1:1000), respectively. The membranes were incubated overnight at 4 °C and then with the secondary antibodies for 2 h at room temperature. After washing with TBST thrice, electrogenerated chemiluminescence (ECL) was used to develop and fix the samples. A gel imager (FluorChem M, ProteinSimple, USA) was used for imaging and semi-quantitative analysis.

### *In vitro* experiment

#### H9c2 cell culture and treatment

H9c2 cells were cultured in 10% foetal bovine serum (FBS; Sigma, USA), penicillin (100 U/mL)/streptomycin (100 U/mL) mixture in Dulbecco’s modified Eagle medium (DMEM) under 37 °C and 5% CO_2_. The H9c2 cells were inoculated into 6-well plates and divided into 5 groups: (1) Control group (10% normal serum), (2) Model group (1 μM DOX (2 mL) was added, and the intervention lasted for 24 h), (3) SMI group (Before modelling, SMI (2 mL) was added and intervene for 24 h, and then SMI was removed, 1 μM DOX (2 mL) was added to each well and intervene for 24 h), (4) ML385 group (30 min before modelling, 1 μM ML385 (2 mL) was added for intervention, and then ML385 was removed, 1 μM DOX (2 mL) was added to each well and intervene for 24 h), (5) SMI + ML385 group (Before modelling, SMI (2 mL) was added and intervene for 24 h, and SMI was removed, then 1 μM ML385 (2 mL) was added for intervention, and then ML385 was removed, 1 μM DOX (2 mL) was added to each well and intervene for 24 h).

### Screening for DOX incubation time

H9c2 cell (density: 1.2 × 10^5^ cells/well) were cultured in DMEM (10% FBS) for different incubation times (12, 24, 36, and 48 h), and induced with 1 μM of DOX. 20 µL of MTT solution (5 mg/mL) was added at 37 °C for 4 h, then removed the medium and 150 µL of DMSO was added. The absorbance value at 490 nm was set in the microplate analyser.

### Screening for the concentration of SMI

H9c2 cells (density: 1.0 × 10^5^ cells/well) were cultured in DMEM (10% FBS) for 24 h, and induced 1 μM of DOX. The effects of different concentrations of SMI (15, 30, 60, 90, 120, and 240 μL/L) on H9c2 cell viability were investigated. MTT solution (20 µL) (5 mg/mL) was added at 37 °C for 4 h, then removed the medium and 150 µL of DMSO was added. The absorbance value at 490 nm was set in the microplate analyser.

### Determination of enzymology parameters in H9c2 cell supernatant

All procedures for CK, LDH, and MDA content and SOD activity detection were performed using the methods presented in the *in vivo* sections.

### Hoechst 33342 stain apoptosis assay

Apoptosis was assessed through observation of morphologic changes in cell nuclei stained with Hoechst 33342 and examined under fluorescence microscopy.

### RT-PCR for mRNA expression

For each group, a cell suspension was inoculated in a 6-well plate at 1 × 10^6^ cells/well. Next, RT-qPCR was performed using the methods presented in *in vivo* RT-PCR.

### Western blot for protein expression

Western blot for protein expression was performed using the methods presented in *in vivo*. The concentrations of antibody were Nrf2 (1:1,000), Keap1 (1:1,000), HO-1 (1:1,000), caspase-3 (1:500), Bax(1:500), Bcl2 (1:500) and β-actin (1:1000), which were visualised through electrochemiluminescence, with the detected protein bands captured on a chemiluminescence image analyser.

### Statistical analysis

All values were presented as mean ± standard deviation (SD). All statistical data were analysed using SPSS (version 23.0) and GraphPad Prism (version 5.0), with the significance level set at *p* < 0.05. Multiple groups were compared using one-way analysis of variance (ANOVA) and the least significant difference method.

## Results

### Docking results

**Figure 1.**
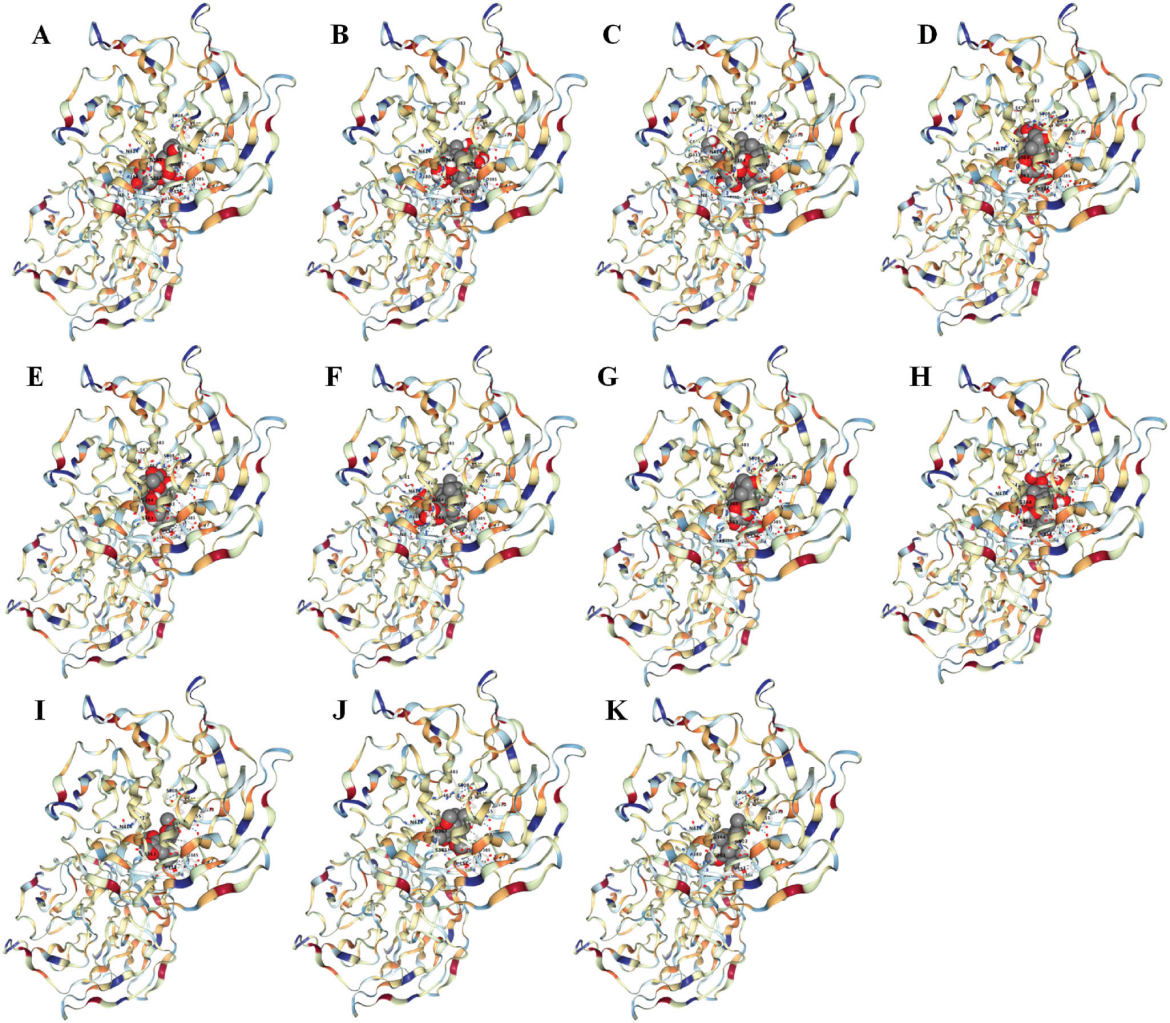
3D diagram of the main components of SMI with Keap-1. (A) Ophiopogonin D, (B) Ginsenoside Rg1, (C) Ginsenoside Rc, (D) Ginsenoside Re, (E) Ginsenoside Rg2, (F) Ginsenoside Rb1, (G) Ginsenoside Rf, (H) Ginsenoside Rd, (I) Schizandrin B, (J) Schisandrol B, (K) Schisandrin.

Molecular docking results showed that the main components of SMI have a good binding ability with Keap-1. In our docking results, ophiopogonin D had the strongest binding ability with Keap-1, followed by ginsenoside Rg1 and ginsenoside Rc (Table 2 and Figure 1). Molecular docking only predicted that SMI might act on Keap-1 and its related signalling pathways, which was then verified by *in vitro* and *in vivo* experiments.

**Table 2.** Docking of main components of SMI with Keap-1.

Chemicals	Vina score	Cavity score	Centre (x, y, z)	Size (x, y, z)
Ophiopogonin D	−10.9	8498	1, −4, −26	35, 35, 35
Ginsenoside Rg1	−9.0	8498	1, −4, −26	35, 35, 35
Ginsenoside Rc	−9.0	8498	1, −4, −26	30, 30, 30
Ginsenoside Re	−8.9	8498	1, −4, −26	35, 35, 35
Ginsenoside Rg2	−8.9	8498	1, −4, −26	35, 35, 35
Ginsenoside Rb1	−8.8	8498	1, −4, −26	35, 35, 35
Ginsenoside Rf	−8.6	8498	1, −4, −26	35, 35, 35
Ginsenoside Rd	−8.5	8498	1, −4, −26	35, 35, 35
Schizandrin B	−8.5	8498	1, −4, −26	35, 35, 35
Schisandrol B	−7.9	8498	1, −4, −26	35, 35, 35
Schisandrin	−7.2	8498	1, −4, −26	35, 35, 35

### *In vivo* experimental results

#### HE staining

**Figure 2.**
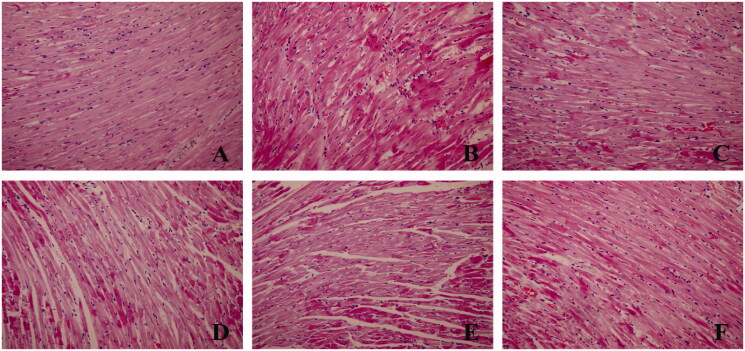
Effects of SMI on the pathological morphology of myocardial tissue (HE × 200). (A) Normal group; (B) Model group; (C) Dex group; (D) SMI-L group; (E) SMI-M group; (F) SMI-H group.

The HE results showed that the myocardial tissue was stained evenly, and the myocardial cells were arranged orderly and the structure was clear in the normal group. The myocardium was broken, loose, wavy and disorganised, the cell morphology was blurred, and the fibroblasts were significantly increased, accompanied by inflammatory cell infiltration and myoplasmic dissolution of myocardial cells in the model group. The fibres were arranged neatly, the cell arrangement was more regular, the fibroblasts were significantly reduced, and inflammatory cell infiltration was small in SMI each dose group and Dex group (Figure 2). The results showed that DOX can induce myocardial tissue damage in rats, SMI and dexrazoxane pre-treatment can significantly improve the pathological changes of myocardial tissue with DOX-induced myocardial injury.

#### Masson staining

**Figure 3.**
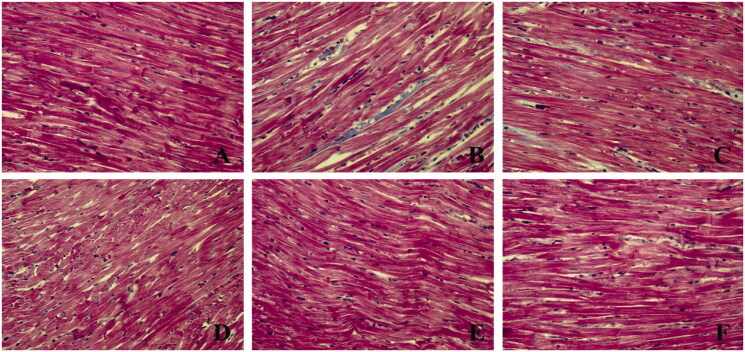
Effect of SMI on the pathological morphology of myocardial tissue (Masson ×400). (A) Normal group; (B) Model group; (C) Dex group; (D) SMI-L group; (E) SMI-M group; (F) SMI-H group.

The Masson results showed that the muscle fibres were red and the collagen fibres were blue. The myocardium of the rats in the normal group was arranged neatly, and there were only a few collagen fibres in the interstitium, and the distribution was relatively even. In the model group, the arrangement of the myocardium was chaotic, the interstitial collagen was significantly increased, and the distribution was disordered. The collagen of myocardial tissue in rats pre-treated with SMI or dexrazoxane was significantly reduced, and the myocardial fibrosis was significantly improved and tended to normalise (Figure 3). The results showed that DOX caused a significant increase in myocardial interstitial fibrosis in model rats, which can be reversed by SMI or dexrazoxane.

#### SMI inhibited the apoptosis of myocardial tissue in model rats

**Figure 4.**
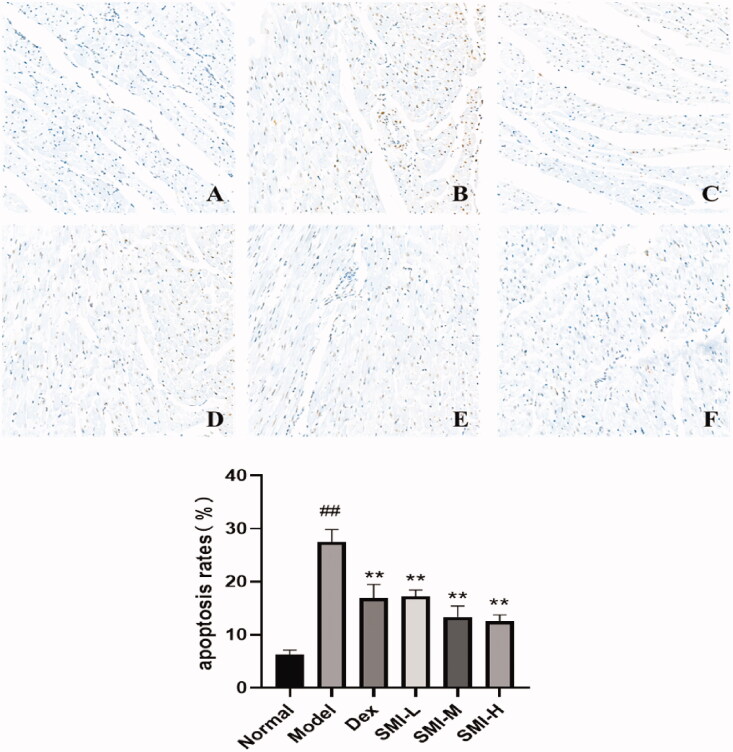
Changes of cardiomyocyte apoptosis rate in model rats. (A) Normal group; (B) Model group; (C) Dex group; (D) SMI-L group; (E) SMI-M group; (F) SMI-H group ^##^*p* < 0.01, vs. Normal group; **p < 0.01, vs. Model group.

After TUNEL staining, the nucleus of normal cardiomyocytes was blue-green, and the nucleus of apoptotic cells was dark brown. A small amount of cell apoptosis occurred in myocardial tissue, and the muscle bundles were arranged neatly and the intercellular space was even in the normal group. The apoptosis was obvious, and the arrangement of myocardial cell muscle bundles was relatively disordered, and the apoptosis rate increased significantly in the model group. The number of dark brown nuclei in myocardial tissue in SMI each dose group and Dex group were significantly reduced, the arrangement of muscle bundles was relatively orderly, and the apoptosis rate was significantly reduced (Figure 4).

#### Effect of SMI on the serum enzyme index in DOX-induced myocardial injury

**Figure 5.**
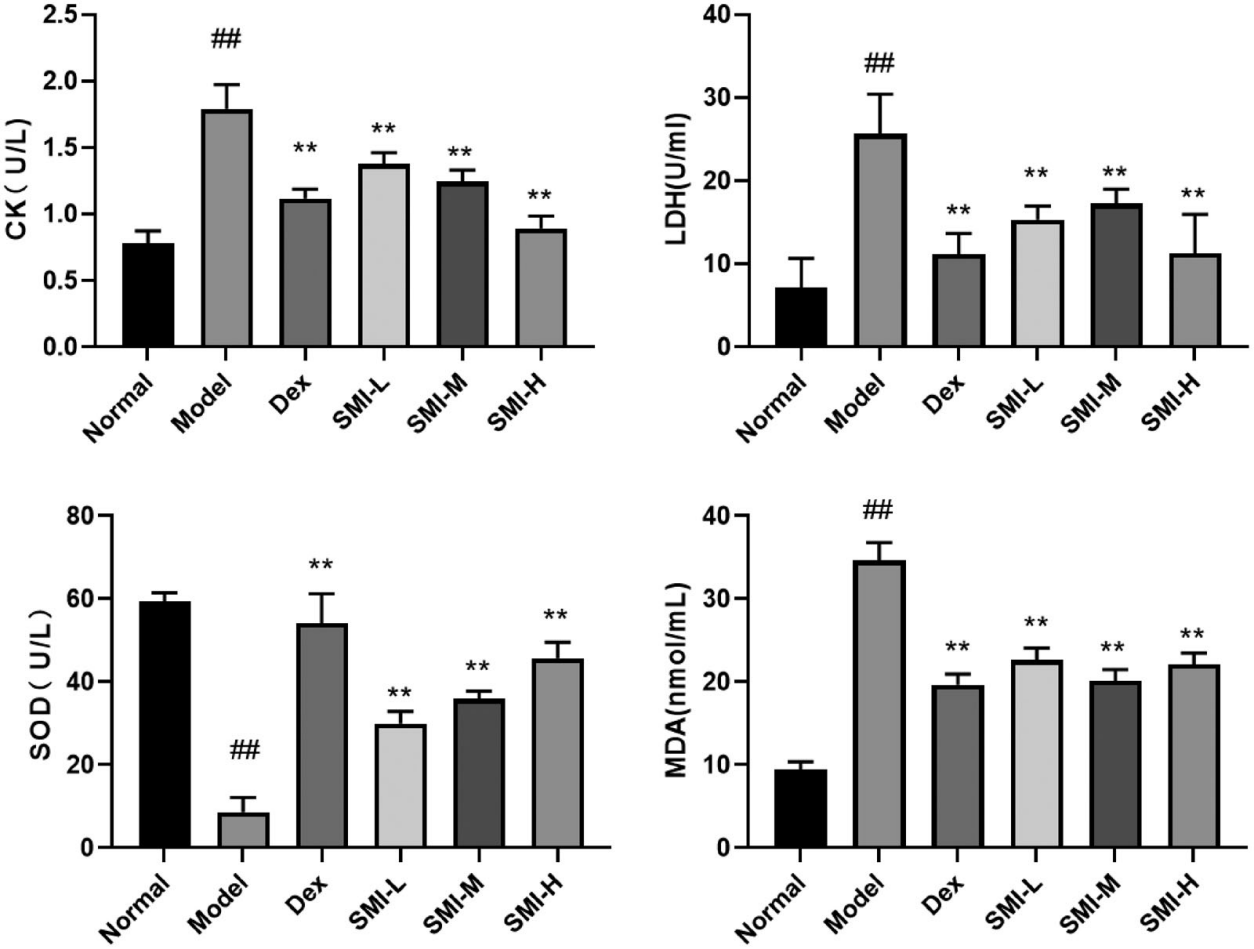
Effect of SMI on the serum enzyme index in DOX-induced myocardial injury. The values were expressed as the mean ± SD (*n* = 10) ^##^*p* < 0.01, vs. Normal group; **p < 0.01, vs. Model group.

The determination of CK and LDH activity can be used for auxiliary diagnosis of myocardial diseases, and reflect the degree of heart damage under pathological conditions. The changes in MDA and SOD activity can reflect the degree of oxidative stress damage to myocardial tissue. Compared with the normal group, the activity of CK, LDH and MDA in the serum of the model group were significantly increased, while the activity of SOD was decreased. And CK activity, LDH and MDA content in serum were significantly reduced in SMI each dose group and Dex group. SOD activity was significantly increased in SMI in each dose group and Dex group (Figure 5). The results show that DOX can induce heart damage and oxidative stress damage in rats. SMI and dexrazoxane could significantly alleviate heart damage, and reduce oxidative stress damage.

#### Effects of SMI on gene expression of Nrf2/Keap1 signalling pathway

**Figure 6.**
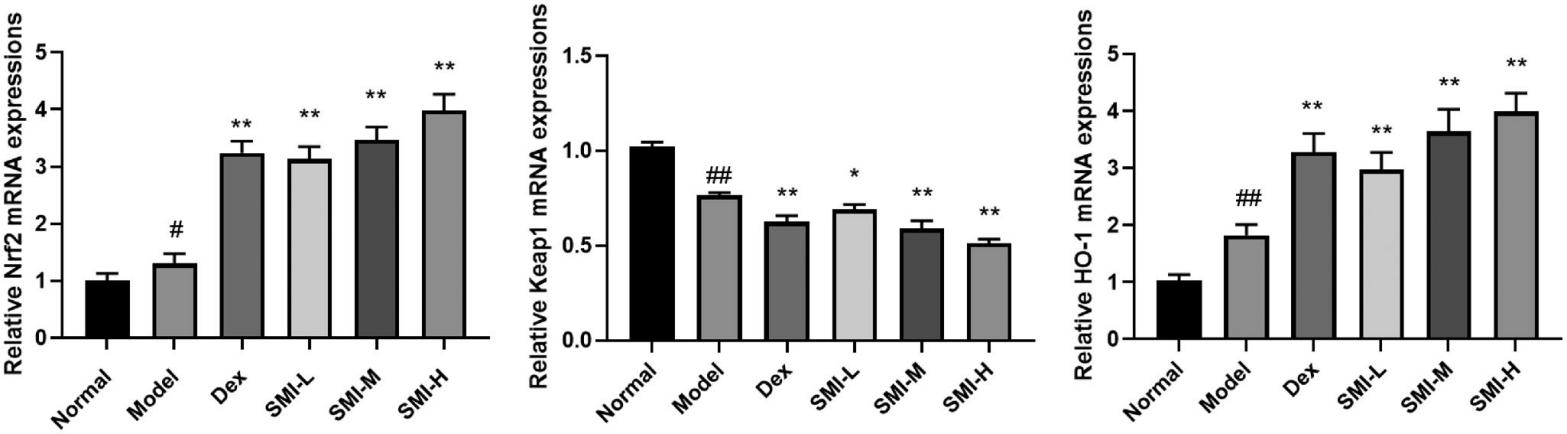
Effects of SMI on gene expression of Nrf2/Keap1 signalling pathway in model rats. ^#^*p* < 0.05, ^##^*p* < 0.01, vs. Normal group; *p < 0.05, **p < 0.01, vs. Model group.

RT-qPCR results showed that the expression of Nrf2 and HO-1 genes were increased and the expression of Keap1 genes was decreased in the model group. Compared with the model group, the expression of Nrf2 and HO-1 gene was increased and the expression of Keap1 gene was decreased in SMI each dose group and Dex group (Figure 6). SMI pre-treatment can significantly activate Nrf2/Keap1 signalling pathway-related gene expression in the myocardium of DOX model rats.

#### Effects of SMI on protein expression of Nrf2/Keap1 signalling pathway

**Figure 7.**
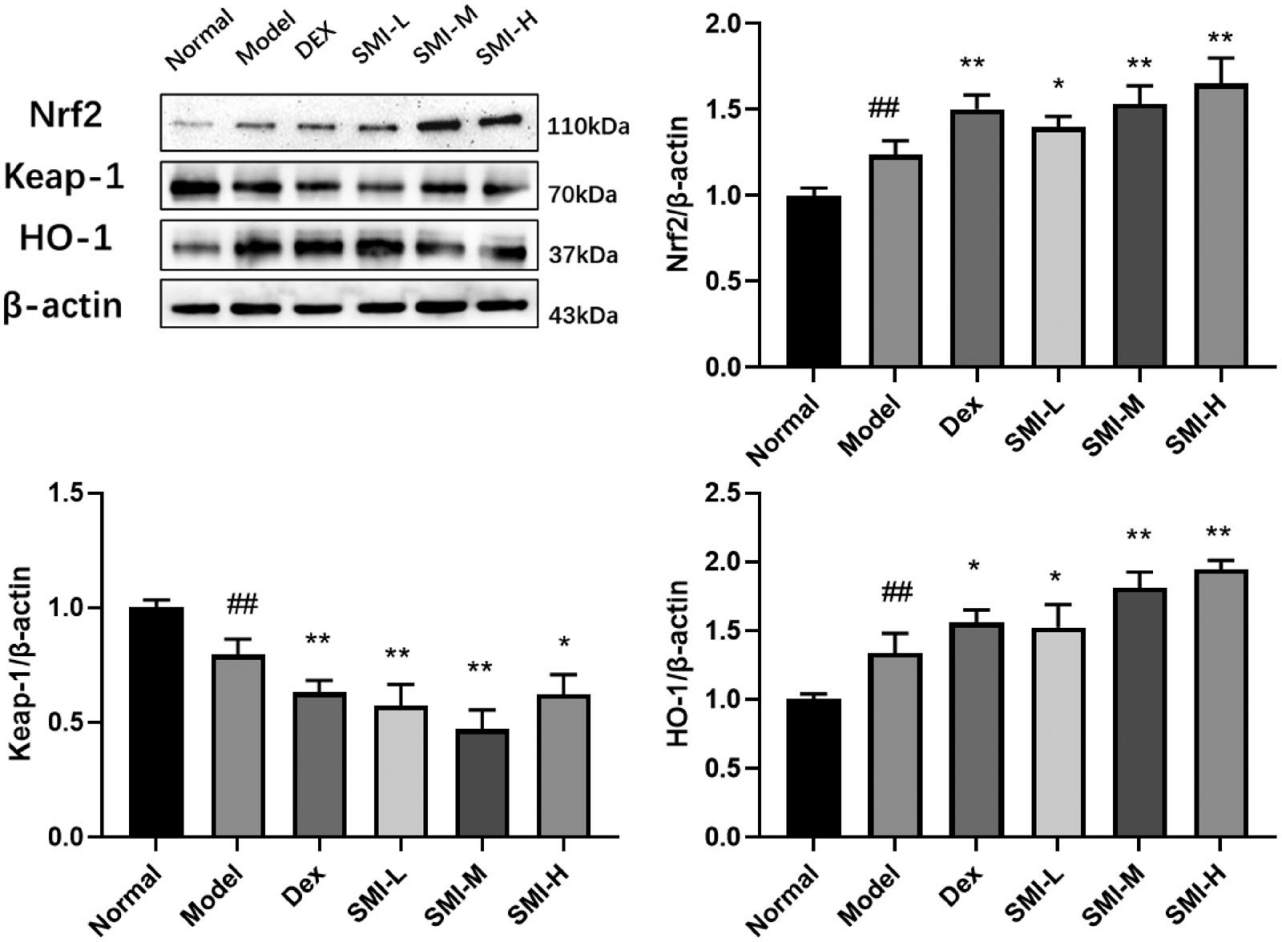
Effects of SMI on protein expression of Nrf2/Keap1 signalling pathway in model rats. (A) Normal group; (B) Model group; (C) Dex group; (D) SMI-L group; (E) SMI-M group; (F) SMI-H group ^##^*p* < 0.01, vs. Normal group; *p < 0.05, **p < 0.01, vs. Model group.

Western blot results showed that the expression of Nrf2 and HO-1 protein were increased and the expression of Keap1 protein was decreased in the model group compared with the normal group. The expression of Nrf2 and HO-1 protein were increased and the expression of Keap1 was decreased in SMI each dose group and dexrazoxane group (Figure 7).

### *In vitro* experimental results

#### Effects of DOX incubation time on the survival rate of H9c2 cells

**Figure 8.**
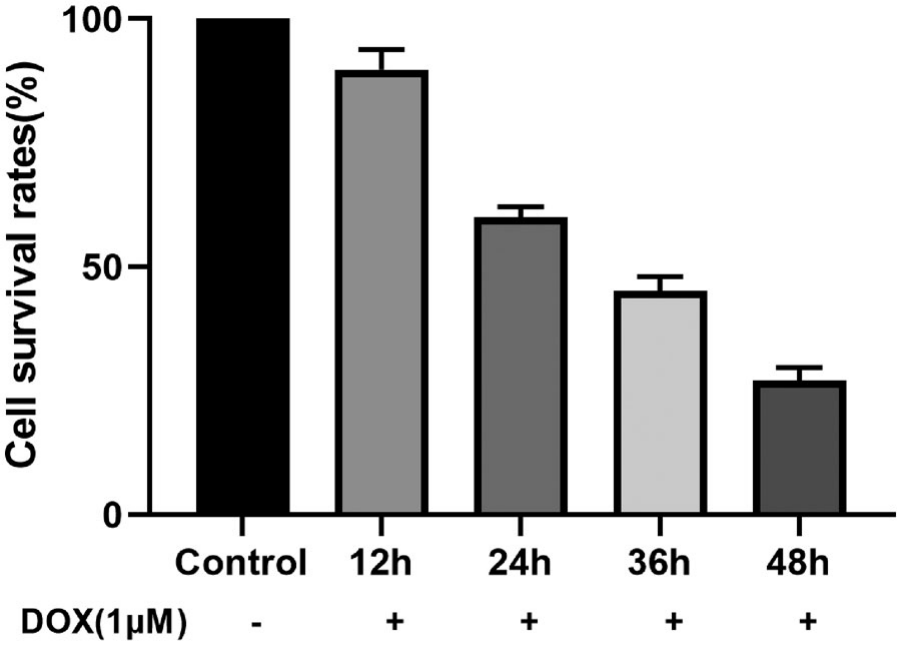
Effects of different incubation times of DOX on the survival rate of H9c2 cells.

MTT assay showed that 1 μM DOX could interfere with H9c2 cardiomyocytes for 12, 24, 36, 48 h, the cell survival rates were significantly decreased. The cell survival rate was 59.92% under 1 μM DOX incubated for 24 h, therefore, subsequent experiments choose this condition as the optimal condition (Figure 8).

#### Effects of different concentrations of SMI on H9c2 cells

**Figure 9.**
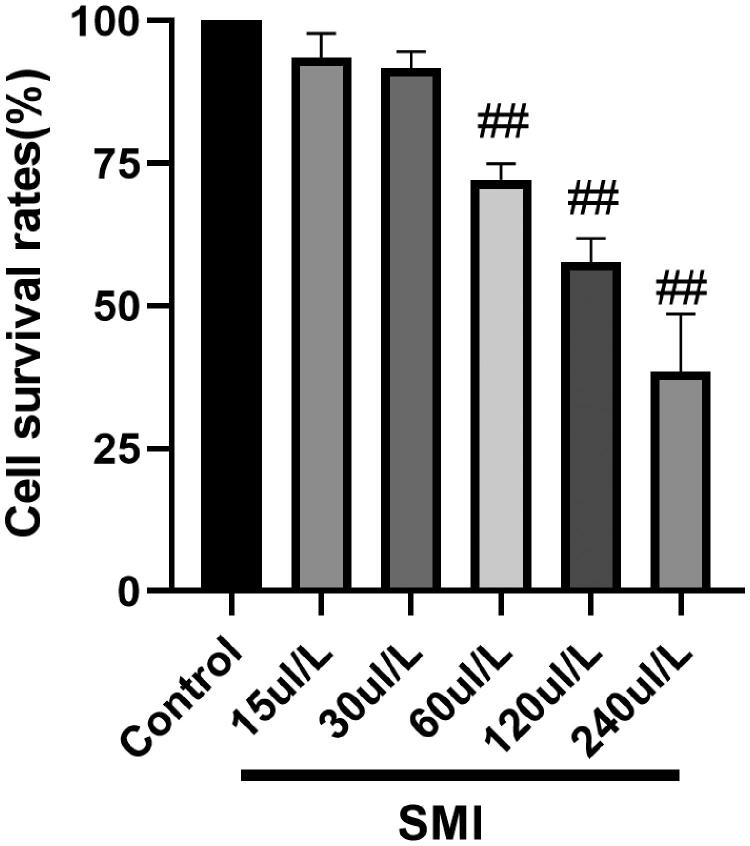
Effects of different concentrations of SMI on H9c2 cells ^##^*p* < 0.01, vs. Control group.

The MTT method was used to determine the optimal concentration of SMI given to H9c2 cells. The concentrations of SMI were 60, 120, 240 μL/L, and the cell survival rate was reduced, which was significantly different from the normal group, indicating that there is cytotoxicity. At the concentrations of 15 and 30 μL/L, the survival rate of H9c2 cells incubated with SMI was not significantly different from that of the control group, indicating that there was no cytotoxic effect (Figure 9). The concentration of SMI is 30 μL/L, and the cell survival rate is above 90%. Therefore, 30 μL/L of SMI was used for cell intervention.

#### Effect of SMI and Nrf2 inhibitor on serum enzymology of H9c2 cells induced by DOX

**Figure 10.**
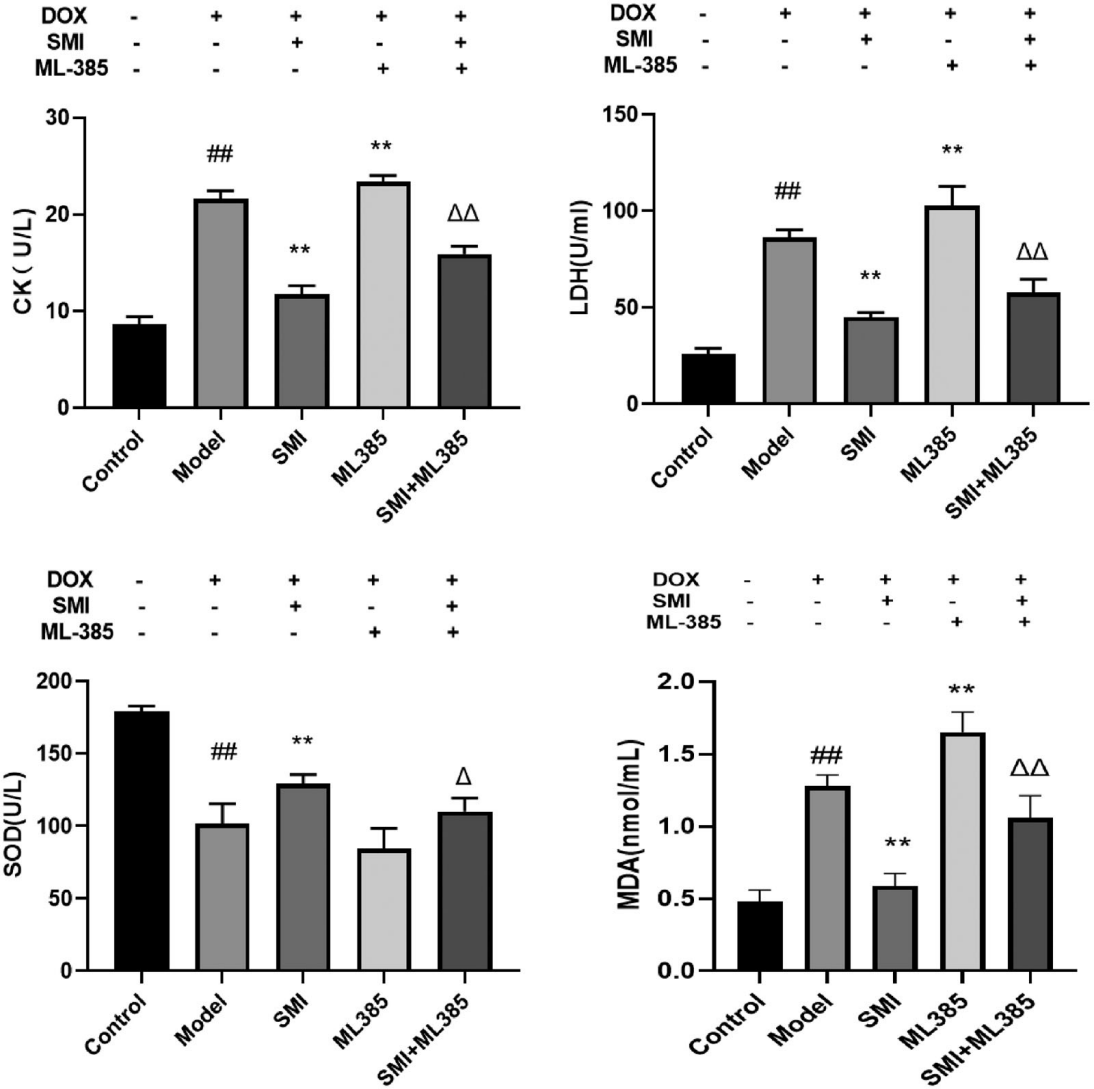
Effect of SMI and Nrf2 inhibitor on serum enzymology of H9c2 cells induced by DOX ^##^*p* < 0.01, vs. Control group; **p < 0.01, vs. Model group; ^Δ^*p* < 0.05, ^ΔΔ^*p* < 0.01, vs. SMI group.

Compared with the Control group, the activity of CK, LDH and MDA of the model group were significantly increased, while the activity of SOD was decreased. CK activity, LDH and MDA content were significantly reduced in the SMI group. SOD activity was significantly increased in the SMI group. Compared with the model group, the activity of CK, LDH and MDA of the ML385 group were significantly increased, while the activity of SOD was decreased. Compared with the SMI group, the activity of CK, LDH and MDA of the SMI + ML385 group were significantly increased, while the activity of SOD was decreased (Figure 10). The results show that SMI could improve DOX-induced cardiomyocyte damage while blocking Nrf2 can significantly reverse the effect of SMI in improving DOX-induced cardiomyocyte damage and significantly aggravate DOX-induced cardiomyocyte damage. Nrf2 is a key regulatory target for SMI to regulate DOX-induced cardiomyocyte damage.

#### Hoechst staining

**Figure 11.**
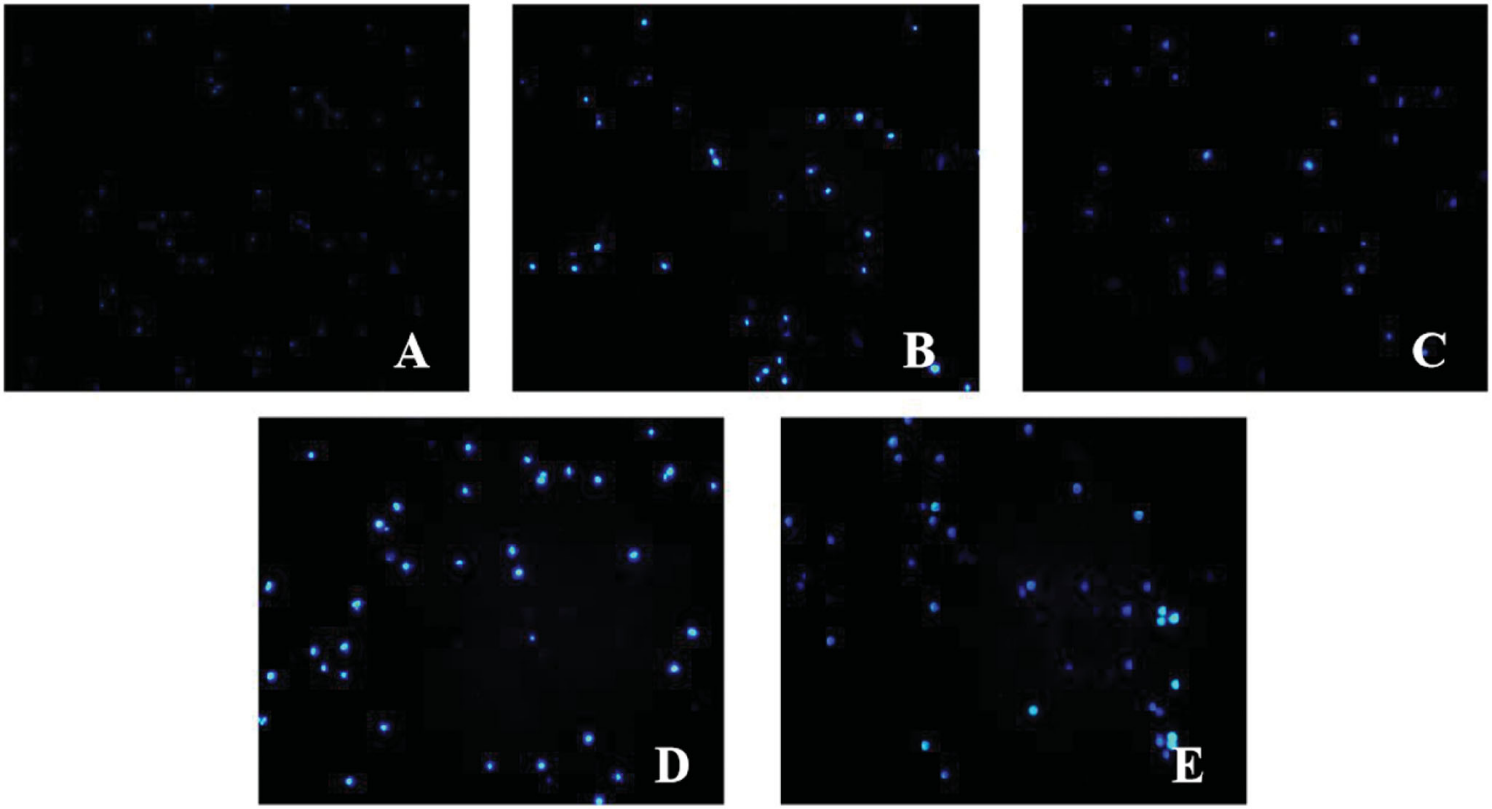
Cell Morphology by Hoechst staining (A) Control group; (B) Model group; (C) SMI group; (D) ML385 group; (E) SMI + ML385 group.

As shown in Figure 11, blue represents the nucleus of H9c2 cells. Apoptosis can be manifested as cell nucleus fragmentation, and bright fluorescence under the microscope. Compared with the control group, the apoptosis of the model group was increased. Compared with the model group, cell apoptosis in the SMI group was reduced. Compared with the SMI group, cell apoptosis increased in the SMI + ML385 group.

#### Effects of SMI on gene expression of Nrf2/Keap1 signalling pathway in H9c2 cells

**Figure 12.**
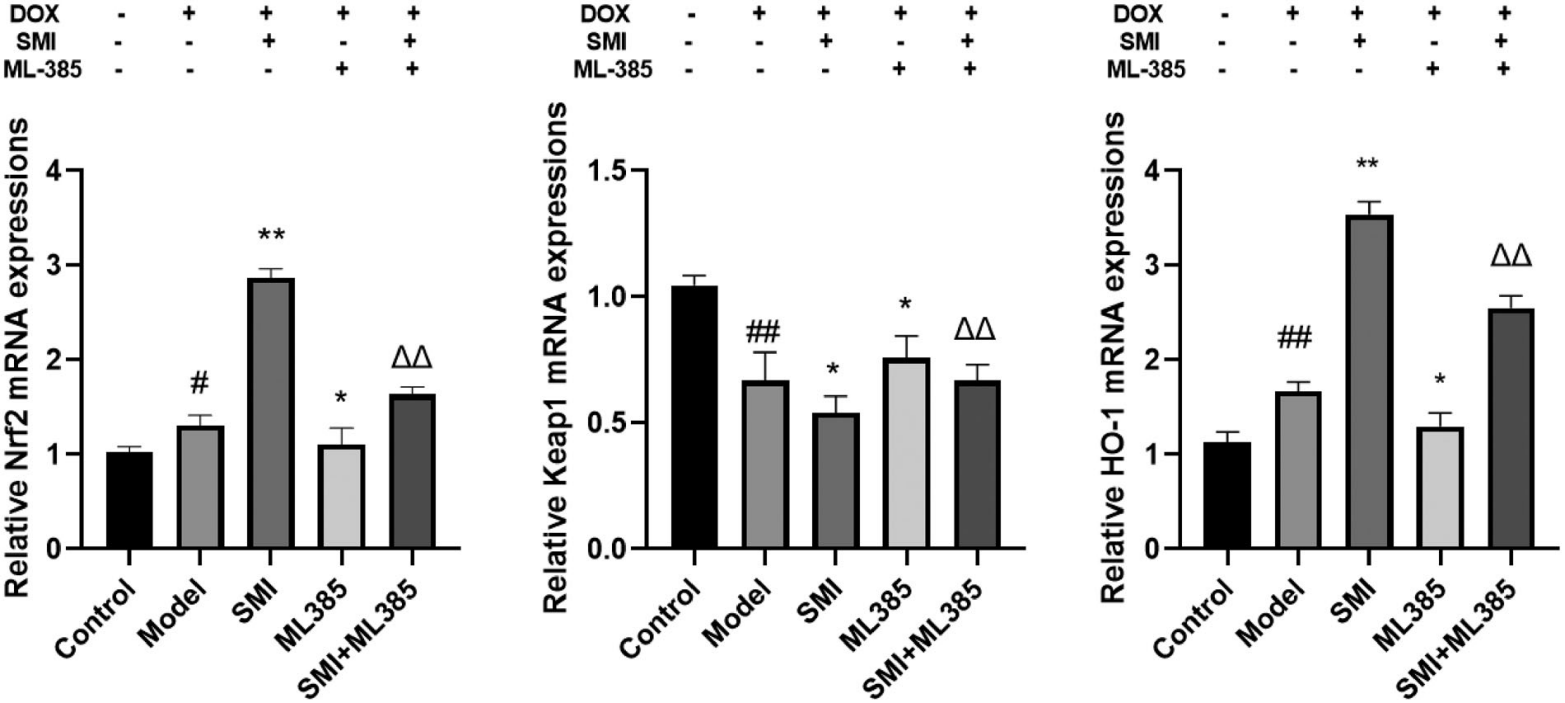
Effects of SMI on gene expression of Nrf2/Keap1 signalling pathway in H9c2 cells. ^#^*p* < 0.05, ^##^*p* < 0.01, vs. Control group; *p < 0.05, **p < 0.01, vs. Model group; ^ΔΔ^*p* < 0.01, vs. SMI group.

Compared with the Control group, the gene expression of Keap1 in the model group was significantly reduced, and the gene expression of Nrf2 and HO-1 was significantly increased. Compared with the Model group, the SMI group can significantly reduce the gene expression of Keap1, and the gene expression of Nrf2 and HO-1 significantly increase. Compared with the Model group, the ML385 group can significantly increase the gene expression of Keap1, and significantly decrease the gene expression of Nrf2 and HO-1. Compared with the SMI group, the SMI + ML385 group can inhibit the gene expression of Nrf2, HO-1 and increase the gene expression of Keap1 (Figure 12). The results show that SMI can significantly inhibit the excessive activation of the Nrf2-Keap1 signalling pathway that DOX induces H9c2 cell damage.

#### SMI improves the expression of Nrf2, Keap1, HO-1 protein in H9c2 cells

**Figure 13.**
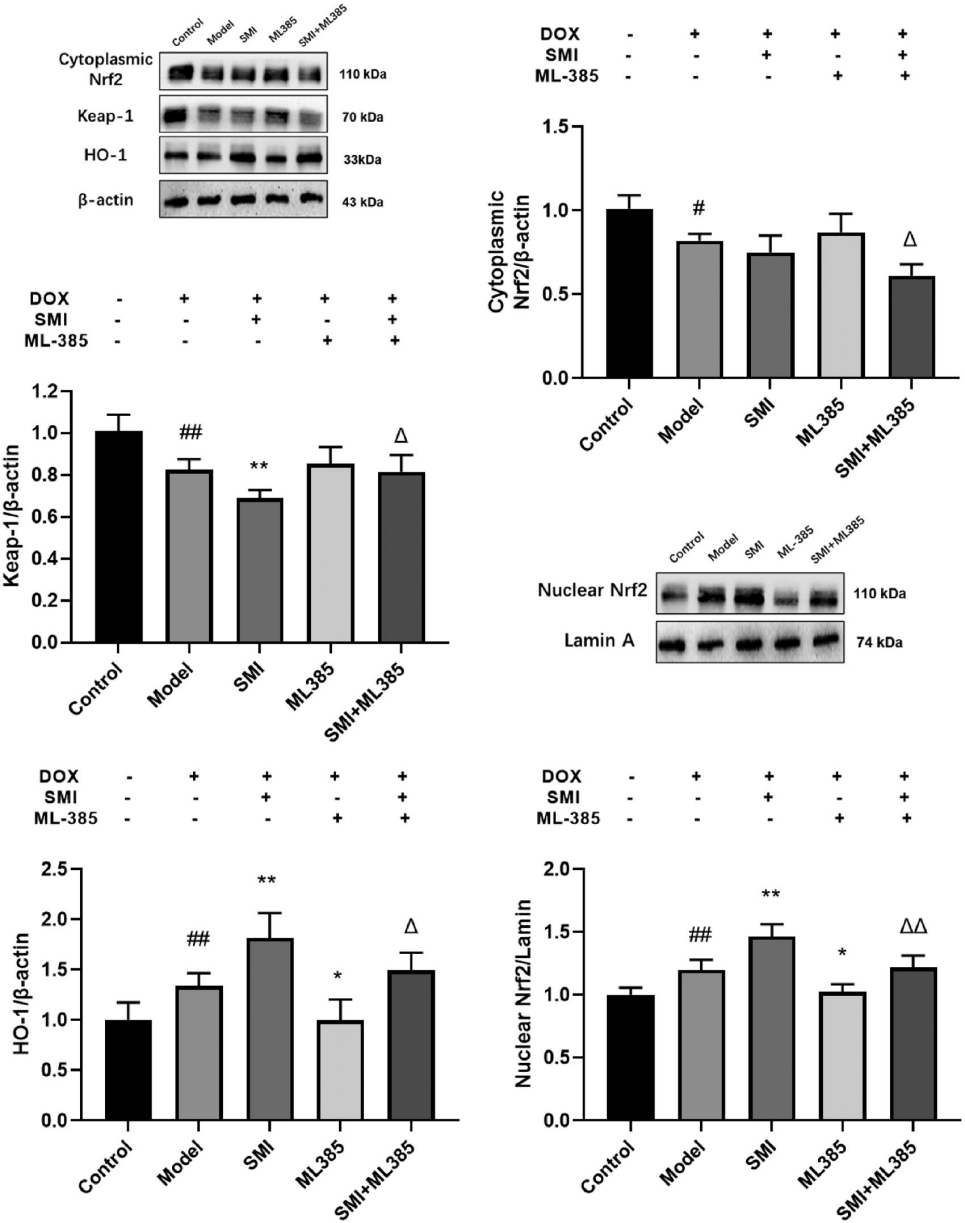
SMI improves the expression of Nrf2, Keap1, HO-1 protein in H9c2 cells. ^#^*p* < 0.05, ^##^*p* < 0.01, vs. Control group; **p < 0.01, vs. Model group; ^Δ^*p* < 0.05, ^ΔΔ^*p* < 0.01, vs. SMI group.

Compared with the control group, the protein expressions of nuclear Nrf2 and HO-1 in the model group were increased, and the protein expression of cytoplasmic Nrf2 and Keap1 were decreased. Compared with the Model group, SMI group can significantly increase the protein expression of nuclear Nrf2 and HO-1, and significantly reduce the protein expression of Keap1. Compared with the Model group, ML385 group can reduce the protein expression of nuclear Nrf2 and HO-1, and increase the protein expression of Keap1. Compared with the SMI group, the SMI + ML385 group can inhibit the expression of protein nuclear Nrf2 and HO-1, and increase the protein expression of Keap1 (Figure 13). The results show that SMI can inhibit the expression of cytosolic Nrf2 and Keap1 protein in H9c2 cells, increase the protein expression of nuclear Nrf2 and HO-1, and significantly regulate DOX-induced overactivation of the Nrf2-Keap1 signalling pathway.

#### SMI improves the protein expression of casepase-3, Bax and Bcl-2 in H9c2 cells

**Figure 14.**
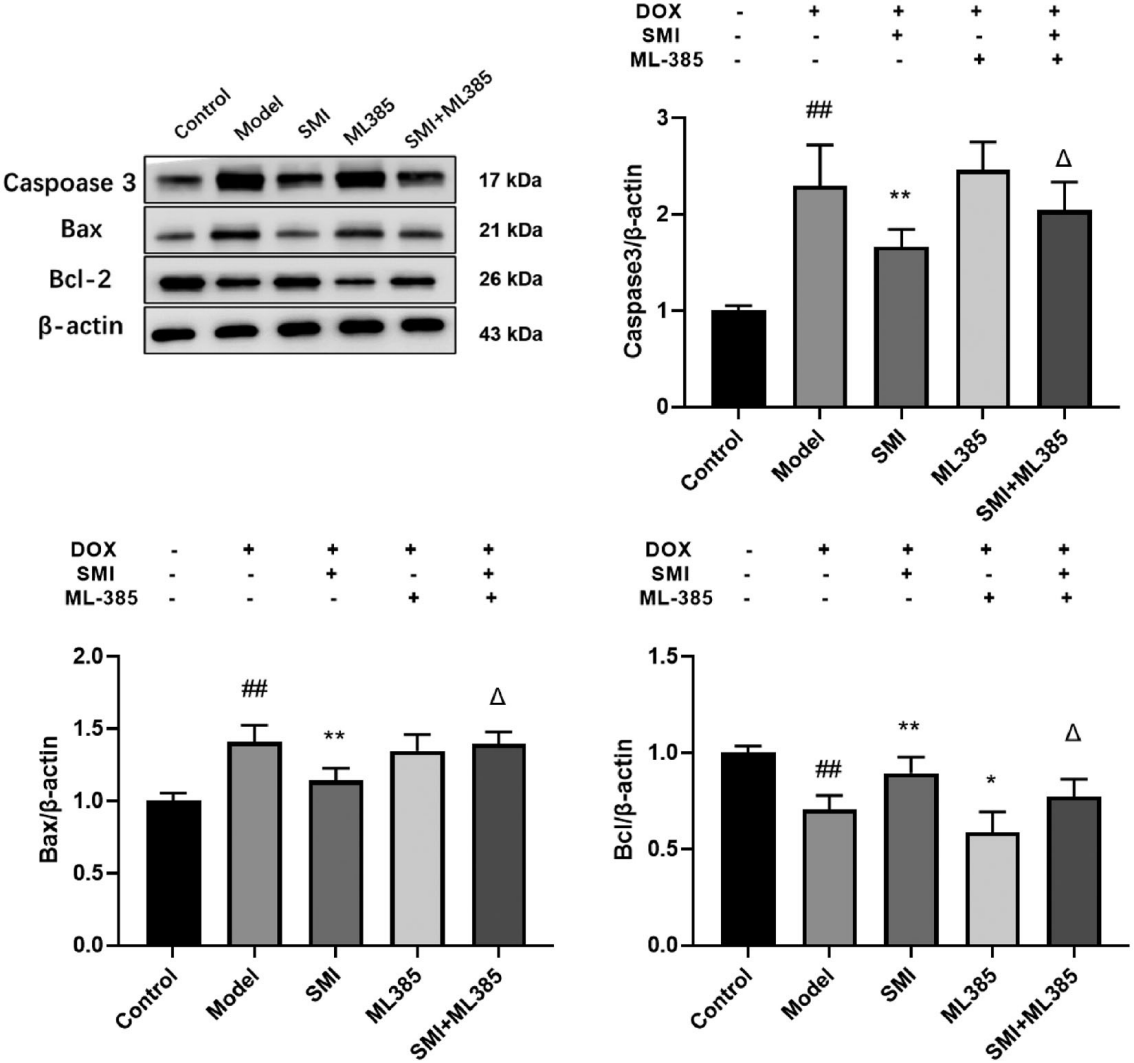
SMI improves the protein expression of casepase-3, Bax and Bcl-2 in H9c2 cells. ^##^*p* < 0.01, vs. Control group; *p < 0.05, **p < 0.01, vs. Model group; ^Δ^*p* < 0.05, vs. SMI group.

In comparison to the Control group, caspase-3 and Bax protein expression increased dramatically in the model group, while Bcl-2 protein expression decreased significantly. Compared with the Model group, the protein expression of Bcl-2 can be significantly increased, and the protein expression of caspase-3 and Bax can be significantly reduced, ML385 group can significantly reduce the protein expression of Bcl-2, and the protein expressions of caspase-3 significantly increase. Compared with the SMI group, the SMI + ML385 group can inhibit the expression of Bcl-2 and increase the protein expression of caspase-3 and Bax (Figure 14). The results show that SMI can inhibit the protein expression of caspase-3 and Bax, increase the protein expression of Bcl-2, and decrease cell apoptosis.

## Discussion

The mechanism of cardiotoxicity induced by anthracyclines is not clear, but it is believed to be related to oxidative stress, inflammatory reaction, apoptosis and autophagy (Fabiani et al. 2021; Rawat et al. 2021). After anthracyclines chelate the ferroptosis, triggers the generation of oxygen free radicals, especially hydroxyl free radicals, resulting in lipid peroxidation of myocardial cell membrane and damage of myocardial mitochondria (Rocca et al. 2020). Therefore, ferroptosis-mediated production of reactive oxygen species (ROS) and the promotion of oxidative stress in the myocardium are important factors of cardiotoxicity induced by anthracycline. Ferroptosis-metabolism is important in DOX-induced cardiotoxicity, which as an electron donor of H_2_O_2_, participates in the Fenton reaction. Ferroptosis-induced production of ROS, including O^2-^, -OH, H_2_O_2_, etc., is the main cause of acute myocardial cytotoxicity and cell death caused by DOX through the destruction of various molecular components and organelles (Akyuz et al. 2021; Lu et al. 2022).

Nrf2 and Keap1 are part of the main regulation system of cell-protective enzyme genes. The activation of Nrf2/Keap1 signalling pathway can effectively maintain cell redox homeostasis, regulate apoptotic proteins, and play an effective anti-inflammatory and anti-apoptotic role (Saha et al. 2020). Nrf2 is a critical regulator of redox homeostasis and cellular antioxidant defences, acting as a negative regulator of oxidative stress by increasing the transcription of antioxidant genes (Kasai et al. 2020). Under homeostatic conditions, Nrf2 is sequestered in the cytoplasm by Keap-1 which interacts with Nrf2 in a redox-sensitive manner (Sui et al. 2020). Under oxidative stress conditions, oxidation of the cysteine residues of Keap1 causes dissociation of Nrf2 from Keap1. Then, Nrf2 is phosphorylated by kinases, is liberated from the Nrf2-Keap1 complex. Subsequently, free Nrf2 is translocated into the nucleus and binds the small Maf transcription factor through the Neh1 domain. The Maf proteins belong to the basic leucine zipper (bZIP) family, which are localised predominantly in the nucleus and interact with DNA. Nrf2/small Maf complex binds to ARE and increases the transcription of ARE-dependent detoxification, antioxidant (SOD and HO-1), and anti-inflammatory genes (Gao et al. 2019; Robledinos-Antón et al. 2019; Gao et al. 2020). HO-1 can maintain redox homeostasis and prevent oxidative stress damage to cardiomyocytes (Jiang et al. 2021). Overexpression of HO-1 can restore the heart failure induced by coronary artery ligation, and knocking out HO-1 can accelerate atherosclerosis. Therefore, regulating Nrf2/Keap1 signalling pathway, inhibiting oxidative stress, maintaining redox homeostasis, reducing myocardial cell apoptosis, and improving myocardial injury is of great significance for the intervention of DOX-induced cardiotoxicity.

DOX is an effective antitumor anthracycline that is widely used to treat ovarian cancer, breast cancer, prostate cancer, hepatocellular carcinoma, and acute lymphoblastic leukaemia. Due to dose-dependent and irreversible cardiac damage, atrial and ventricular arrhythmia, and heart failure, its clinical application is limited (Modesto et al. 2021). Chest pain, palpitations, abnormal electrocardiogram, decreased left ventricular ejection fraction, and alterations in the myocardial enzyme spectrum are all clinical symptoms of DOX-induced cardiotoxicity (Sadek et al. 2020; Che et al. 2021). Therefore, the acute cardiotoxicity rat model induced by DOX can be used in this study. Currently, a large dose of one-time injection of DOX can induce acute cardiotoxicity in rat models, and DOX (15 mg/kg) was injected in a single dose. The results showed that the rats myocardial rupture, disordered arrangement, myocyte sarcoplasmic dissolved, fibroblasts increased accompanied by inflammatory cells infiltration, myocardial enzyme changes, conform to the pathological physiology characteristic of myocardial injury caused by DOX, which showed that the rat model and the choice of the observation time is feasible, can be used in the evaluation of myocardial injury caused by drug prevention and treatment of DOX. Meanwhile, DOX-induced pathological changes in model rats' cardiac tissue, including necrosis, apoptosis, and interstitial fibrosis, and serum CK and LDH levels were dramatically elevated, meeting clinical diagnostic criteria, showing that the model was successfully prepared.

Dexrazoxane is the only FDA-approved cardioprotective medication for DOX. Dexrazoxane may ameliorate cardiotoxicity and protect heart function by inhibiting both apoptosis and necroptosis in cardiomyocyte injury caused by DOX (Zhang et al. 2021). Although dexrazoxane protects the heart from the cardiotoxicity of DOX, it can also produce side effects such as decreased appetite, nausea, and vomiting, myeloid inhibition, neurotoxic phlebitis, and diarrhoea in the clinic (Lue et al. 2018). Dexrazoxane was used as a positive control in this study to assess the effect of SMI on DOX cardiac toxicity. SMI has been shown in recent studies to improve heart function, protect myocardial cells, and increase myocardial energy, as well as to be beneficial in the treatment of heart diseases such as myocardial infarction, myocardial ischaemia-reperfusion injury, heart failure, diabetic cardiomyopathy, and myocarditis (Cao et al. 2020).

In this study, *in vivo* experiment results showed that SMI pre-treatment can significantly reduce the apoptosis and necrosis of myocardial cells, decrease the infiltration of inflammatory cells and interstitial fibrosis of rats. CK and LDH levels are important indicators of clinical myocardial injury, and SMI can significantly reduce CK and LDH levels, implying that pre-treatment with SMI can significantly improve changes in heart pathology and serum enzymology in model rats induced by DOX, and may play a beneficial role in preventing DOX-induced acute cardiac injury. SOD, a critical ROS scavenger, helps maintain the balance of ROS in the body. The activity of SOD is reduced, the accumulation of ROS is increased, and cardiotoxicity will be aggravated in the DOX-induced cardiotoxicity model (Jiang et al. 2020; Tene et al. 2021). MDA, a highly toxic lipid peroxidation, can migrate to both the inside and outside of cells, hence exacerbating the damage range caused by oxidative stress (Wan et al. 2021). MDA also has the potential to destroy the membrane's integrity and result in the leaking of LDH and CK into cells. Therefore, the activity of SOD and MDA may provide insight into the extent of oxidative stress damage in cardiomyocytes. SMI can significantly reduce the activity of MDA and increase the activity of SOD, suggesting that the effect of SMI on DOX cardiac toxicity is related to anti-oxidative stress injury. Nrf2 acts as a protective player against DOX-induced myocardial oxidative stress. SMI has been shown to significantly decrease the expression of the Keap1 gene and protein in myocardial tissue while increasing the expression of the Nrf2 and HO-1 genes and proteins, implying that its effect on heart injury is due to the Nrf2/Keap1 signalling pathway intervention in myocardial tissue and inhibition of oxidative stress injury. The results of *in vitro* experiments indicated that pre-treatment with SMI can significantly inhibit the protein and gene expression of Nrf2, Keap1, and HO-1, promote Nrf2 nucleation, activate the Nrf2/Keap1 antioxidant stress signalling pathway, decrease cardiomyocyte apoptosis, caspase-3 and Bax expression, and increase Bcl-2 expression. SMI protects myocardial cells from DOX-induced damage by activating the Nrf2/Keap1 signalling pathway and anti-oxidative stress injury-induced myocardial cell apoptosis.

## Conclusions

*In vivo* and *in vitro* investigations elucidated the effects of SMI on DOX-induced cardiotoxicity and myocardial damage. The mechanism was revealed to be by interfering with Nrf2-mediated oxidative stress injury, activating Nrf2/Keap1 signalling pathway, resisting oxidative stress injury and inhibiting myocardial cell necrosis and apoptosis. SMI establishes a theoretical foundation for the clinical intervention of DOX cardiotoxicity.

## Data Availability

The data used to support the findings of this study are available from the corresponding author upon request.

## References

[CIT0001] Akyuz E, Doganyigit Z, Eroglu E, Moscovicz F, Merelli A, Lazarowski A, Auzmendi J. 2021. Myocardial iron overload in an experimental model of sudden unexpected death in epilepsy. Front Neurol. 12:609236. 33643194 10.3389/fneur.2021.609236 PMC7905080

[CIT0002] Cao Y, Han X, Pan H, Jiang Y, Peng X, Xiao W, Rong J, Chen F, He J, Zou L, et al. 2020. Emerging protective roles of shengmai injection in septic cardiomyopathy in mice by inducing myocardial mitochondrial autophagy via caspase-3/Beclin-1 axis. Inflamm Res. 69(1):41–50. 31712853 10.1007/s00011-019-01292-2

[CIT0003] Che Y, Wang Z, Yuan Y, Zhou H, Wu H, Wang S, Tang Q. 2021. By restoring autophagic flux and improving mitochondrial function, corosolic acid protects against dox-induced cardiotoxicity. Cell Biol Toxicol. DOI:10.1007/s10565-021-09619-8. 34296331

[CIT0004] Chen R-C, Sun G-B, Ye J-X, Wang J, Zhang M-d, Sun X-B. 2017. Salvianolic acid B attenuates doxorubicin-induced ER stress by inhibiting TRPC3 and TRPC6 mediated Ca^2+^ overload in rat cardiomyocytes. Toxicol Lett. 276:21–30. 28495616 10.1016/j.toxlet.2017.04.010

[CIT0005] Chen Y, Tang Y, Xiang Y, Xie YQ, Huang XH, Zhang YC. 2015. Shengmai injection improved doxorubicin-induced cardiomyopathy by alleviating myocardial endoplasmic reticulum stress and caspase-12 dependent apoptosis. Biomed Res Int. 2015:952671. 25839043 10.1155/2015/952671 PMC4369903

[CIT0006] Chen Q, Zhang P, Xiao QX, Liu Q, Zhang Y. 2021. Protective effect of Shengmai injection on myocardial endothelial cell glycoprotein detachment after myocardial ischemia-reperfusion injury in isolated rat hearts. Perfusion. 36(7):757–765. 33070762 10.1177/0267659120965921

[CIT0007] Dallons M, Schepkens C, Dupuis A, Tagliatti V, Colet JM. 2020. New insights about doxorubicin-induced toxicity to cardiomyoblast-derived H9C2 cells and dexrazoxane cytoprotective effect: contribution of *in Vitro1H-NMR Metabonomics*. Front Pharmacol. 11:79. 32153402 10.3389/fphar.2020.00079 PMC7044126

[CIT0008] Fabiani I, Aimo A, Grigoratos C, Castiglione V, Gentile F, Saccaro LF, Arzilli C, Cardinale D, Passino C, Emdin M. 2021. Oxidative stress and inflammation: determinants of anthracycline cardiotoxicity and possible therapeutic targets. Heart Fail Rev. 26(4):881–890. 33319255 10.1007/s10741-020-10063-9 PMC8149360

[CIT0009] Gao Y, Lv X, Yang H, Peng L, Ci X. 2020. Isoliquiritigenin exerts antioxidative and anti-inflammatory effects via activating the KEAP-1/Nrf2 pathway and inhibiting the NF-κB and NLRP3 pathways in carrageenan-induced pleurisy. Food Funct. 11(3):2522–2534. 32141447 10.1039/c9fo01984g

[CIT0010] Gao HL, Xia YZ, Zhang YL, Yang L, Kong LY. 2019. Vielanin P enhances the cytotoxicity of doxorubicin via the inhibition of PI3K/Nrf2-stimulated MRP1 expression in MCF-7 and K562 DOX-resistant cell lines. Phytomedicine. 58:152885. 31009836 10.1016/j.phymed.2019.152885

[CIT0011] Gu J, Huang H, Liu C, Jiang B, Li M, Liu L, Zhang S. 2021. Pinocembrin inhibited cardiomyocyte pyroptosis against doxorubicin-induced cardiac dysfunction via regulating Nrf2/Sirt3 signaling pathway. Int Immunopharmacol. 95:107533. 33752080 10.1016/j.intimp.2021.107533

[CIT0012] Huang CH, Chang HP, Su SY, Chen WK, Chang YJ, Lee YC, Kuo YJ. 2019. Traditional Chinese medicine is associated with a decreased risk of heart failure in breast cancer patients receiving doxorubicin treatment. J Ethnopharmacol. 229:15–21. 30261193 10.1016/j.jep.2018.09.030

[CIT0013] Jiang G, Chen D, Li W, Liu C, Liu J, Guo Y. 2020. Effects of wogonoside on the inflammatory response and oxidative stress in mice with nonalcoholic fatty liver disease. Pharm Biol. 58(1):1177–1183. 33253604 10.1080/13880209.2020.1845747 PMC7875554

[CIT0014] Jiang Y, Liu Y, Xiao W, Zhang D, Liu X, Xiao H, You S, Yuan L. 2021. Xinmailong attenuates doxorubicin-induced lysosomal dysfunction and oxidative stress in H9c2 cells via HO-1. Oxid Med Cell Longev. 2021:5896931. 33854694 10.1155/2021/5896931 PMC8019640

[CIT0015] Jnoff E, Albrecht C, Barker JJ, Barker O, Beaumont E, Bromidge S, Brookfield F, Brooks M, Bubert C, Ceska T, et al. 2014. Binding mode and structure-activity relationships around direct inhibitors of the Nrf2-Keap1 complex. ChemMedChem. 9(4):699–705. 24504667 10.1002/cmdc.201300525

[CIT0016] Kasai S, Shimizu S, Tatara Y, Mimura J, Itoh K. 2020. Regulation of Nrf2 by mitochondrial reactive oxygen species in physiology and pathology. Biomolecules. 10(2):320. 10.3390/biom10020320 PMC7072240 32079324

[CIT0017] Li L, Li J, Wang Q, Zhao X, Yang D, Niu L, Yang Y, Zheng X, Hu L, Li Y. 2020. Shenmai injection protects against doxorubicin-induced cardiotoxicity via maintaining mitochondrial homeostasis. Front Pharmacol. 11:815. 32581790 10.3389/fphar.2020.00815 PMC7289952

[CIT0018] Li Y, Ruan X, Xu X, Li C, Qiang T, Zhou H, Gao J, Wang X. 2019. Shengmai injection suppresses angiotensin II-induced cardiomyocyte hypertrophy and apoptosis via activation of the AMPK signaling pathway through energy-dependent mechanisms. Front Pharmacol. 10:1095. 31616303 10.3389/fphar.2019.01095 PMC6764192

[CIT0019] Liu Y, Grimm M, Dai WT, Hou MC, Xiao ZX, Cao Y. 2020. CB-Dock: a web server for cavity detection-guided protein-ligand blind docking. Acta Pharmacol Sin. 41(1):138–144. 31263275 10.1038/s41401-019-0228-6 PMC7471403

[CIT0020] Liu X, Tan W, Yang F, Wang Y, Yue S, Wang T, Wang X. 2018. Shengmai injection reduces apoptosis and enhances angiogenesis after myocardial ischaemia and reperfusion injury in rats. Biomed Pharmacother. 104:629–636. 29803176 10.1016/j.biopha.2018.04.180

[CIT0021] Lu H, Xiao H, Dai M, Xue Y, Zhao R. 2022. Britanin relieves ferroptosis-mediated myocardial ischaemia/reperfusion damage by upregulating GPX4 through activation of AMPK/GSK3β/Nrf2 signalling. Pharm Biol. 60(1):38–45. 34860639 10.1080/13880209.2021.2007269 PMC8648013

[CIT0022] Lue Y, Gao C, Swerdloff R, Hoang J, Avetisyan R, Jia Y, Rao M, Ren S, Atienza V, Yu J, et al. 2018. Humanin analog enhances the protective effect of dexrazoxane against doxorubicin-induced cardiotoxicity. Am J Physiol Heart Circ Physiol. 315(3):H634–H643. 29775411 10.1152/ajpheart.00155.2018 PMC6734085

[CIT0023] Modesto PN, Polegato BF, Dos Santos PP, Grassi LDV, Molina LCC, Bazan SGZ, Pereira EJ, Fernandes AAH, Fabro AT, Androcioli VN, et al. 2021. Green tea (*Camellia sinensis*) extract increased topoisomerase IIβ, improved antioxidant defense, and attenuated cardiac remodeling in an acute doxorubicin toxicity model. Oxid Med Cell Longev. 2021:1–10. 10.1155/2021/8898919 PMC8116148 34035878

[CIT0024] Prathumsap N, Shinlapawittayatorn K, Chattipakorn SC, Chattipakorn N. 2020. Effects of doxorubicin on the heart: from molecular mechanisms to intervention strategies. Eur J Pharmacol. 866:172818. 31758940 10.1016/j.ejphar.2019.172818

[CIT0025] Rawat PS, Jaiswal A, Khurana A, Bhatti JS, Navik U. 2021. Doxorubicin-induced cardiotoxicity: an update on the molecular mechanism and novel therapeutic strategies for effective management. Biomed Pharmacother. 139:111708. 34243633 10.1016/j.biopha.2021.111708

[CIT0026] Robledinos-Antón N, Fernández-Ginés R, Manda G, Cuadrado A. 2019. Activators and inhibitors of NRF2: a review of their potential for clinical development. Oxid Med Cell Longev. 2019:9372182. 31396308 10.1155/2019/9372182 PMC6664516

[CIT0027] Rocca C, Pasqua T, Cerra MC, Angelone T. 2020. Cardiac damage in anthracyclines therapy: focus on oxidative stress and inflammation. Antioxid Redox Signal. 32(15):1081–1097. 31928066 10.1089/ars.2020.8016

[CIT0028] Sadek KM, Mahmoud SFE, Zeweil MF, Abouzed TK. 2020. Proanthocyanidin alleviates doxorubicin-induced cardiac injury by inhibiting NF-κB pathway and modulating oxidative stress, cell cycle, and fibrogenesis. J Biochem Mol Toxicol. 35:e22716. 10.1002/jbt.22716 33484087

[CIT0029] Saha S, Buttari B, Panieri E, Profumo E, Saso L. 2020. An overview of Nrf2 signaling pathway and its role in inflammation. Molecules. 25(22):5474. 10.3390/molecules25225474 PMC7700122 33238435

[CIT0030] Sawicki KT, Sala V, Prever L, Hirsch E, Ardehali H, Ghigo A. 2021. Preventing and treating anthracycline cardiotoxicity: new insights. Annu Rev Pharmacol Toxicol. 61:309–332. 33022184 10.1146/annurev-pharmtox-030620-104842

[CIT0032] Sui YB, Zhang KK, Ren YK, Liu L, Liu Y. 2020. The role of Nrf2 in astragaloside IV-mediated antioxidative protection on heart failure. Pharm Biol. 58(1):1192–1198. 33253607 10.1080/13880209.2020.1849319 PMC7717863

[CIT0033] Tene K, Kalyan Kumar M, Basveshwar G, Eswara Rao P, Jagadeesh Kumar G, Kumar P, Pemmaraju DB, Murty U, Gogoi R, Naidu V. 2021. Polyphenolic-rich compounds from *Dillenia pentagyna* (Roxb.) attenuates the doxorubicin-induced cardiotoxicity: a high-frequency ultrasonography assisted approach. Front Pharmacol. 12:624706. 34079455 10.3389/fphar.2021.624706 PMC8166202

[CIT0034] Wan Y, He B, Zhu D, Wang L, Huang R, Zhu J, Wang C, Gao F. 2021. Nicotinamide mononucleotide attenuates doxorubicin-induced cardiotoxicity by reducing oxidative stress, inflammation and apoptosis in rats. Arch Biochem Biophys. 712:109050. 34610336 10.1016/j.abb.2021.109050

[CIT0035] Wang Y, Zhou X, Chen X, Wang F, Zhu W, Yan D, Shang H. 2020. Efficacy and safety of shengmai injection for chronic heart failure: a systematic review of randomized controlled trials. Evid Based Complement Alternat Med. 2020:9571627. 32655670 10.1155/2020/9571627 PMC7322585

[CIT0036] Zhan S, Shao Q, Fan X, Li Z. 2015. Development of a sensitive LC-MS/MS method for simultaneous quantification of eleven constituents in rat serum and its application to a pharmacokinetic study of a Chinese medicine Shengmai injection. Biomed Chromatogr. 29(2):275–284. 25043947 10.1002/bmc.3273

[CIT0037] Zhang H, Wang Z, Liu Z, Du K, Lu X. 2021. Protective effects of dexazoxane on rat ferroptosis in doxorubicin-induced cardiomyopathy through regulating HMGB1. Front Cardiovasc Med. 8:685434. 34336950 10.3389/fcvm.2021.685434 PMC8318065

[CIT0038] Zhang X, Zhang J, Ji X, Wei Z, Ding B, Liu G, Lv X, Zheng Y, Zhan S. 2021. A quantitative serum proteomic analysis helps to explore the comprehensive mechanism and identify serum biomarkers of Shengmai injection's effect on isoproterenol-induced myocardial ischemia in rats. Front Pharmacol. 12:666429. 33995093 10.3389/fphar.2021.666429 PMC8113823

[CIT0039] Zhu J, Hu Q, Shen S. 2020. Enhanced antitumor efficacy and attenuated cardiotoxicity of doxorubicin in combination with lycopene liposomes. J Liposome Res. 30(1):37–44. 30741056 10.1080/08982104.2019.1580720

[CIT0040] Zhu J, Ye Q, Xu S, Chang YX, Liu X, Ma Y, Zhu Y, Hua S. 2019. Shengmai injection alleviates H2O2‑induced oxidative stress through activation of AKT and inhibition of ERK pathways in neonatal rat cardiomyocytes. J Ethnopharmacol. 239:111677. 30615921 10.1016/j.jep.2019.01.001

